# Multi-institutional study of GRE scores as predictors of STEM PhD degree completion: GRE gets a low mark

**DOI:** 10.1371/journal.pone.0206570

**Published:** 2018-10-29

**Authors:** Sandra L. Petersen, Evelyn S. Erenrich, Dovev L. Levine, Jim Vigoreaux, Krista Gile

**Affiliations:** 1 Department of Veterinary and Animal Sciences, University of Massachusetts Amherst, Amherst, Massachusetts, United States of America; 2 School of Graduate Studies, Rutgers, The State University of New Jersey, New Brunswick, New Jersey, United States of America; 3 Graduate School, University of New Hampshire, Durham, New Hampshire, United States of America; 4 Department of Biology and Office of the Provost, University of Vermont, Burlington, Vermont, United States of America; 5 Department of Mathematics and Statistics, University of Massachusetts Amherst, Amherst, Massachusetts, United States of America; Northwestern University, UNITED STATES

## Abstract

The process of selecting students likely to complete science, technology, engineering and mathematics (STEM) doctoral programs has not changed greatly over the last few decades and still relies heavily on Graduate Record Examination (GRE) scores in most U.S. universities. It has been long debated whether the GRE is an appropriate selection tool and whether overreliance on GRE scores may compromise admission of students historically underrepresented in STEM. Despite many concerns about the test, there are few studies examining the efficacy of the GRE in predicting PhD completion and even fewer examining this question in STEM fields. For the present study, we took advantage of a long-lived collaboration among institutions in the Northeast Alliance for Graduate Education and the Professoriate (NEAGEP) to gather comparable data on GRE scores and PhD completion for 1805 U.S./Permanent Resident STEM doctoral students in four state flagship institutions. We found that GRE Verbal (GRE V) and GRE Quantitative (GRE Q) scores were similar for women who completed STEM PhD degrees and those who left programs. Remarkably, GRE scores were significantly higher for men who left than counterparts who completed STEM PhD degrees. In fact, men in the lower quartiles of GRE V or Q scores finished degrees more often than those in the highest quartile. This pattern held for each of the four institutions in the study and for the cohort of male engineering students across institutions. GRE scores also failed to predict time to degree or to identify students who would leave during the first year of their programs. Our results suggests that GRE scores are not an effective tool for identifying students who will be successful in completing STEM doctoral programs. Considering the high cost of attrition from PhD programs and its impact on future leadership for the U.S. STEM workforce, we suggest that it is time to develop more effective and inclusive admissions strategies.

## Introduction

Advances in science, technology, engineering and mathematics (STEM) fields drive innovation and economic progress in the U.S. and globally. Thus, the selection and training of doctoral students who will become leaders in these disciplines has widespread and long-term consequences. Approximately 18,000 U.S. students currently earn doctoral degrees in STEM fields (excluding social and behavioral sciences) annually [1], but that number represents only around 59% of the entering cohort [2]. Numerous factors may account for this low level of completion, and considerable resources have been directed towards identifying and remediating these factors [2]. Nevertheless, in view of high societal, institutional and personal costs of this attrition, it may be time to also reassess how STEM doctoral programs select students for admission.

Reassessment is timely because STEM doctoral education is rapidly changing to better prepare students for working in teams to solve complex environmental, medical and societal problems. Increasingly, didactic classroom learning and individual project completion are being replaced with problem-based learning and collaborative, interdisciplinary research [3–5]. During this evolution in STEM training, the graduate admissions process has not changed correspondingly. It still relies quite heavily on the Graduate Record Examination (GRE) and cut-off scores [6, 7], despite recommendations of the ETS [8]. Unfortunately, the GRE does not measure creativity, problem-solving abilities or other characteristics viewed as important for success in graduate school [9–11]. Another consideration is that GRE scores are generally lower for women and non-Asian minorities (American Indians, Hawaiian/Pacific Islanders, Black/African Americans, Mexican American, Puerto Ricans and other Hispanics) [12], groups currently earning the fewest STEM doctorates [13]. This is a looming problem because women earn only 25% of STEM PhDs but make up more than 50% of the U.S. population [1, 14]. Similarly non-Asian minority groups currently comprise nearly 33% of the population [15] and earn only around 9% of the STEM PhD degrees [1]. Thus, relying on GRE scores to select students can limit diversity in STEM doctoral programs [16, 17] and could result in a shortage of STEM leaders in the future.

Considering the wide ranging and long-term ramifications of relying on the GRE in STEM doctoral admissions decisions, there are surprisingly few studies on the efficacy of this examination to predict the most important measure of success—completion of PhD degrees. One of the largest studies, a meta-analysis of 1753 independent studies conducted over 50 years [18], found negative or very weak correlations between GRE scores and degree completion in life sciences and in math-physical sciences. Others [10] argued that meta-analyses of multiple studies are limited by what the authors of the primary papers choose to study, and require data adjustments for comparability, as well as tools to estimate unreported data. For these reasons, Burton and Wang [10] used a common design to simultaneously collect data from four participating institutions. In addition, they analyzed data from masters and doctoral students independently, and data from chemistry, mathematics and psychology students separately from those in English. Despite these refinements, Burton and Wang also failed to find evidence of a relationship between GRE scores and STEM PhD completion. However, while they analyzed data from over 1300 students, only 340 were PhD students in science-related disciplines. In addition, issues regarding the small percentage of students who had earned PhD degrees by the time data were collected made their findings somewhat difficult to interpret.

Despite caveats with the larger studies described above, results were consistent with more recent studies from biomedical doctoral programs housed in individual institutions. Researchers at the Ponce Health Sciences University Biomedical Sciences Program found that GRE scores did not differentiate students who left the program from those who were retained [19]. In the umbrella biomedical PhD program at the University of North Carolina Chapel Hill, GRE Verbal (GRE V) and Quantitative (GRE Q) scores were similar regardless of whether students completed degrees in less than 5 years, in 5–6 years, in greater than 6 years or if they withdrew [20]. Similarly, neither GRE V nor GRE Q scores were correlated with PhD degree attainment in the Vanderbilt University Medical School’s biomedical umbrella program [21]. Overall, these findings suggest that GRE scores are not useful for identifying students who will complete PhD degrees in biomedical research programs.

In the present study, we sought to determine whether GRE scores are predictive of PhD completion in a broader range of STEM fields and whether there are gender differences in the predictive abilities of the GRE. To obtain sufficient data for meaningful comparisons, we collected information on over 1800 students from four variously sized state flagship research universities that participate in the Northeast Alliance for Graduate Education and the Professoriate. The Alliance is a long-standing collaboration originally funded by the National Science Foundation to diversify STEM PhD programs. We sought to avoid problems encountered in previous multi-institutional studies by including only data from doctoral students who had enrolled in STEM programs (listed below). In addition, we focused only on U.S. citizens or Permanent Residents to reduce confounding variables. An analysis of a somewhat larger dataset that included social science students from the NEAGEP cohort was made available previously [22].

## Materials and methods

This research was approved by the University of Massachusetts Amherst Institutional Review Board (Approval number 2018–4724). No informed consent request was required because data were analyzed anonymously. All identifiable data are stored on a password-protected computer in the possession of the PI.

### Sample

Four state flagship research universities with graduate student enrollments ranging from approximately 1,500 to 14,000 provided data for all U.S. citizens and Permanent Residents entering PhD programs in STEM between 2000 and 2005. This date range ensured that students had started their programs at least 10 years before the time of data collection. The sample included 1805 students of whom 57.5% were men and 42.5% women.

The STEM fields included in this study were Biological Sciences, Physical Sciences, Chemical Sciences, Computer and Information Sciences, Engineering, Geosciences, Mathematical Sciences and related technology areas. The data collected included GRE V and GRE Q scores, major/department, year of entry to the program, whether degree was completed, year of program completion or withdrawal, gender, and race/ethnicity (the small sample size precluded use of race/ethnicity data in the present study). All GRE scores used in this study were from the pre-2011 version of the test and ranged from 200 to 800. Each institution entered its data on a standardized template. To ensure comparability, data from each institution were checked and some entries were removed if students were still enrolled after 10 years, lacked GRE scores or were not in the STEM fields listed above.

### Analysis

We used a logistic regression approach to model degree completion as a function of institution, gender and GRE V or GRE Q scores, including all interactions in the analysis. When interactions were found to be insignificant in explaining variability in degree completion we did not consider them in subsequent analyses. Gender differences in GRE scores for each institution were also examined across the four institutions using two-way ANOVA.

We used two-way ANOVA to compare means of GRE V or GRE Q scores between men and women who completed or left STEM doctoral programs within the first year. To determine whether there were gender differences in the rate of leaving during the first year, we used contingency tables and Chi-Square tests.

For a more in-depth analysis, we divided subjects into quartiles based on rank-ordered GRE V or GRE Q scores and combined quartile data across institutions. We determined the mean time to degree and the completion rate for each gender at each quartile. We used two-way ANOVA to determine whether time to degree varied by quartile for either gender, and compared male and female completion rates using contingency tables and Chi-Square tests. We performed follow-up studies to test whether students with GRE Q scores in the lowest quartile had high GRE V scores that might confer an advantage in graduate school. Mean GRE V scores were calculated for each GRE Q quartile and compared using one-way ANOVA.

We then examined the relationship between GRE Q scores and PhD completion in engineering, a discipline generally considered to be mathematics-intensive. We first used two-way ANOVA with gender and institution as the main effects. Finding no significant institutional effect or interaction between institution and gender on GRE scores, we combined data for all institutions. We used two-way ANOVA to compare GRE scores between men and women and between students in engineering and non-engineering STEM doctoral programs. We also compared completion rates for males and females in engineering using Chi-Squared tests. We then used two-way ANOVA with gender and completion status as main effects to determine whether GRE scores differed significantly between students who completed or left programs. We further evaluated interaction effects using Sidak’s multiple comparison test. We also divided men enrolled in engineering PhD programs into quartiles based on GRE Q scores. We then calculated the percent completion for each quartile and compared rates among quartiles using Chi-Squared tests.

## Results

**Fig 1:**
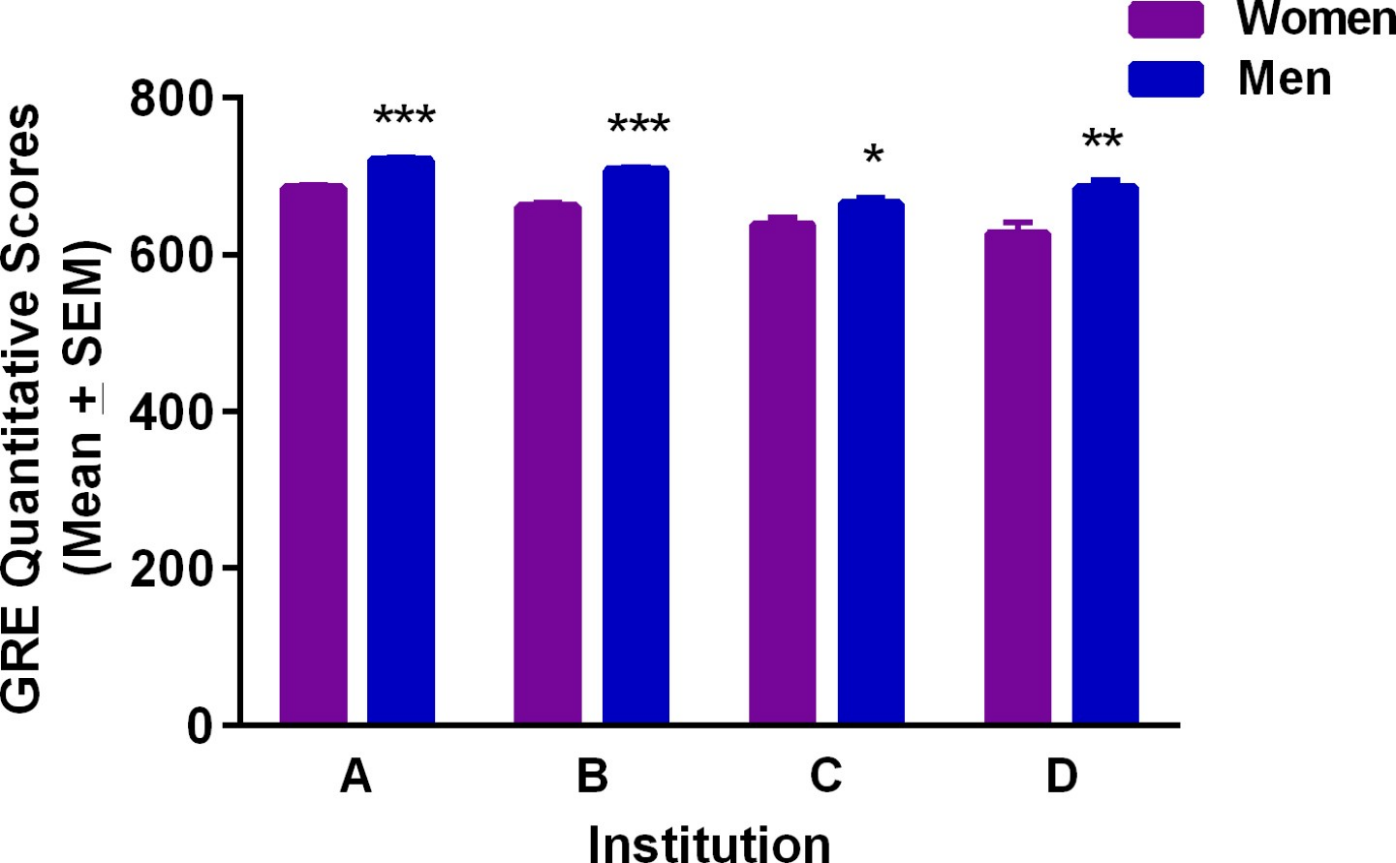
GRE Quantitative scores for STEM PhD students (women and men) in four state flagship universities. *Significantly different from women, p<0.05; **p<0.001; ***p<0.0001.

There were no significant differences in GRE V scores between men and women in any institution (data not shown), but there was a significant gender effect in GRE Q scores that was examined using Tukey’s multiple comparison test *post hoc*. Men had significantly higher GRE Q scores than women in every institution (Fig 1).

Data in Table 1 show that there were no significant gender differences in completion rates, time to degree, time students stayed in programs before leaving, or the percentage who left during the first year. Completion rates for both men and women were similar to the national 10-year completion rate of 59.1% in STEM PhD programs overall [2]. In addition, the average time to degree for students in our study was less than 6 years, and the national 6-year STEM PhD completion rate is only 42.7% [2]. It is also notable that the institutions in our study are all public, but the completion rates were similar to those of private institutions that may have higher aggregate GRE scores [2].

**Table 1:** PhD completion data for men and women in STEM PhD programs.

	Women	Men
**Completed PhD (N)**	450	652
**Left Program (N)**	317	386
**Completion Rate (%)**	58.6	62.8
**Years to Degree (Mean ±** **SEM)**	5.85 ± 0.05	5.93 ± 0.07
**Years Before Leaving (Mean ±** **SEM)**	3.3 ± 0.15	3.1 ± 0.15
**Left During First Year (%)**	6.4	4.5

Table 2 shows results of our analysis of GRE scores and PhD completion for women and men. Our logistic regression analysis found interactions between gender and institution effects were not significant for GRE V or Q scores, but gender and GRE Q interactions were significant.

**Table 2:** GRE Verbal (GRE V), GRE Quantitative (GRE Q) (mean ± SEM) scores for women and men who completed STEM PhD degrees or left without PhD degrees.

		GRE V	GRE Q			GRE V	GRE Q
**Women Who Completed PhD Degrees**	Mean	534.2	671.6	**Men Who Completed PhD Degrees**	Mean	535.7	698.9
	SEM	4.8	4.1		SEM	4.1	3.4
	N	450	450		N	652	652
**Women Who Did Not Complete**	Mean	532.5	666.1	**Men Who Did Not Complete**	Mean	551.6*	722.8***
	SEM	6.0	4.9		SEM	5.5	3.6
	N	317	317		N	386	386

*Significantly higher than scores of men who completed degrees; p<0.05;

*** p<0.0001.

We then used Women as the reference category and determined that there was no effect of GRE Q scores on PhD completion for women. However, the difference between genders was significant so we next used the category Men as the reference and found that GRE Q scores had a negative predictive effect on PhD completion. Our two-way ANOVA verified that GRE scores were not associated with PhD completion for women, and that men who completed STEM PhDs had significantly **lower** GRE Q scores than those who left their programs. We also found significantly lower GRE V scores for men who completed than for those who left programs (Table 2).

As shown in Table 3, we found no differences in GRE V or GRE Q scores between students (women or men) who completed and those who left programs within the first year.

**Table 3:** GRE Verbal (GRE V), GRE Quantitative (GRE Q) (mean ± SEM) scores for women and men who completed STEM PhD degrees or left during the first year.

		GRE V	GRE Q			GRE V	GRE Q
**Women Who Completed PhD Degrees**	Mean	534.2	671.6	**Men Who Completed PhD Degrees**	Mean	535.7	698.9
	SEM	4.8	4.1		SEM	4.1	3.4
	N	450	450		N	652	652
**Women Who Left in First Year**	Mean	515.0	657.1	**Men Who Left in First Year**	Mean	541.0	710.4
	SEM	13.4	11.1		SEM	14.4	13.1
	N	52	52		N	49	49

Data in Table 4 show that there were no significant differences in completion rates for women based on quartile for either GRE V or GRE Q scores. This is despite a difference of approximately 267 points between those in the highest and lowest quartiles of GRE V, and a difference of 225 points between highest and lowest quartiles of GRE Q scores. In contrast to women, men in the **lowest** quartile of GRE Q scores finished at a **higher** rate than counterparts in all three higher quartiles. It is notable that men in the lowest quartile for GRE Q scores averaged 196 points below those of men in the highest quartile, the group with the lowest completion rate. Similarly, men in the third quartile of GRE V scores finished at a significantly higher rate than those in the two higher quartiles.

**Table 4:** Mean (± SEM), range of GRE V and GRE Q scores, percentile into which mean scores fell, range of score percentiles, and completion rates in quartiles of 767 women and 1038 men who enrolled in STEM doctoral programs in four state flagship universities.

	**GRE V**	**Q1 (High)**	**Q2**	**Q3**	**Q4 (Low)**
**Women**	**Mean**	660.8	572.8	505.9	393.6
	**SEM**	3.3	1.2	1.7	4.0
	**Range of Scores**	610–800	550–600	470–540	240–460
	**Percentile of Mean Score**	92	75	58	27
	**Range of Score Percentiles**	85–99	69–83	49–67	1–47
	**Completion (%)**	58.7	63.6	64.2	52.0
	**GRE V**	**Q1 (High)**	**Q2**	**Q3**	**Q4 (Low)**
**Men**	**Mean**	679.5	578.2	510.5	404.6
	**SEM**	2.9	1.3	1.2	3.5
	**Range**	620–800	550–610	480–540	250–470
	**Percentile of Mean Score**	94	77	59	29
	**Range of Score Percentiles**	86–99	69–85	52–67	1–49
	**Completion (%)**	59.4*	59.2*	69.3	62.7

	**GRE Q**	**Q1 (High)**	**Q2**	**Q3**	**Q4 (Low)**
**Women**	**Mean**	776.9	710.7	641.2	551.1
	**SEM**	1.4	1.4	1.3	3.9
	**Range**	750–800	680–740	610–670	290–600
	**Percentile of Mean Score**	86	67	45	27
	**Range of Score Percentiles**	77–94	58–74	38–54	3–35
	**Completion (%)**	62.2	57.1	56.2	59.5
	**GRE Q**	**Q1 (High)**	**Q2**	**Q3**	**Q4 (Low)**
**Men**	**Mean**	792.7	750.3	698.0	597.0
	**SEM**	0.5	0.9	1.1	3.8
	**Range**	780–800	730–770	670–720	200–660
	**Percentile of Mean Score**	91	77	63	34
	**Range of Score Percentiles**	87–94	72–84	54–69	1–51
	**Completion (%)**	56.2***	59.4***	60.6**	74.0

*Significantly lower than completion rate of those in Quartile 3 (Q3); p<0.05.

**Significantly lower than completion rate of Q4 (lowest quartile); p<0.001.

***Significantly lower than Q4, p<0.0001). Percentiles are approximated based on mean GRE scores for each quartile and data provided in [23].

**Fig 2:**
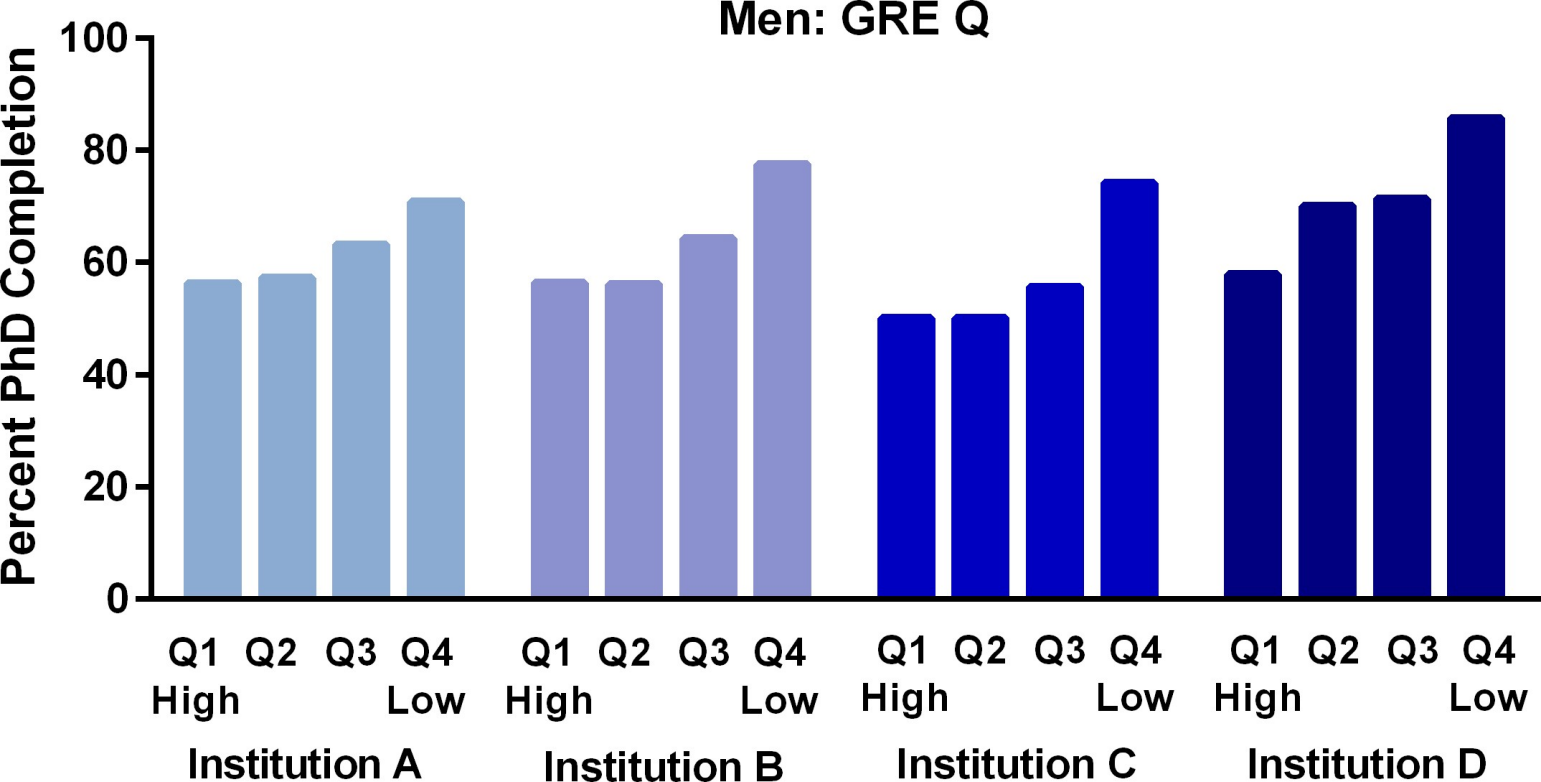
Relationships between GRE Quantitative quartile (Q) scores and PhD completion rates for men in four state flagship universities. Q1 is the highest and Q4 the lowest quartile for the scores.

The pattern of men in the lowest quartile for GRE Q finishing at a higher rate than those in the highest quartile was seen in each of the four institutions in the study (Fig 2).

**Fig 3:**
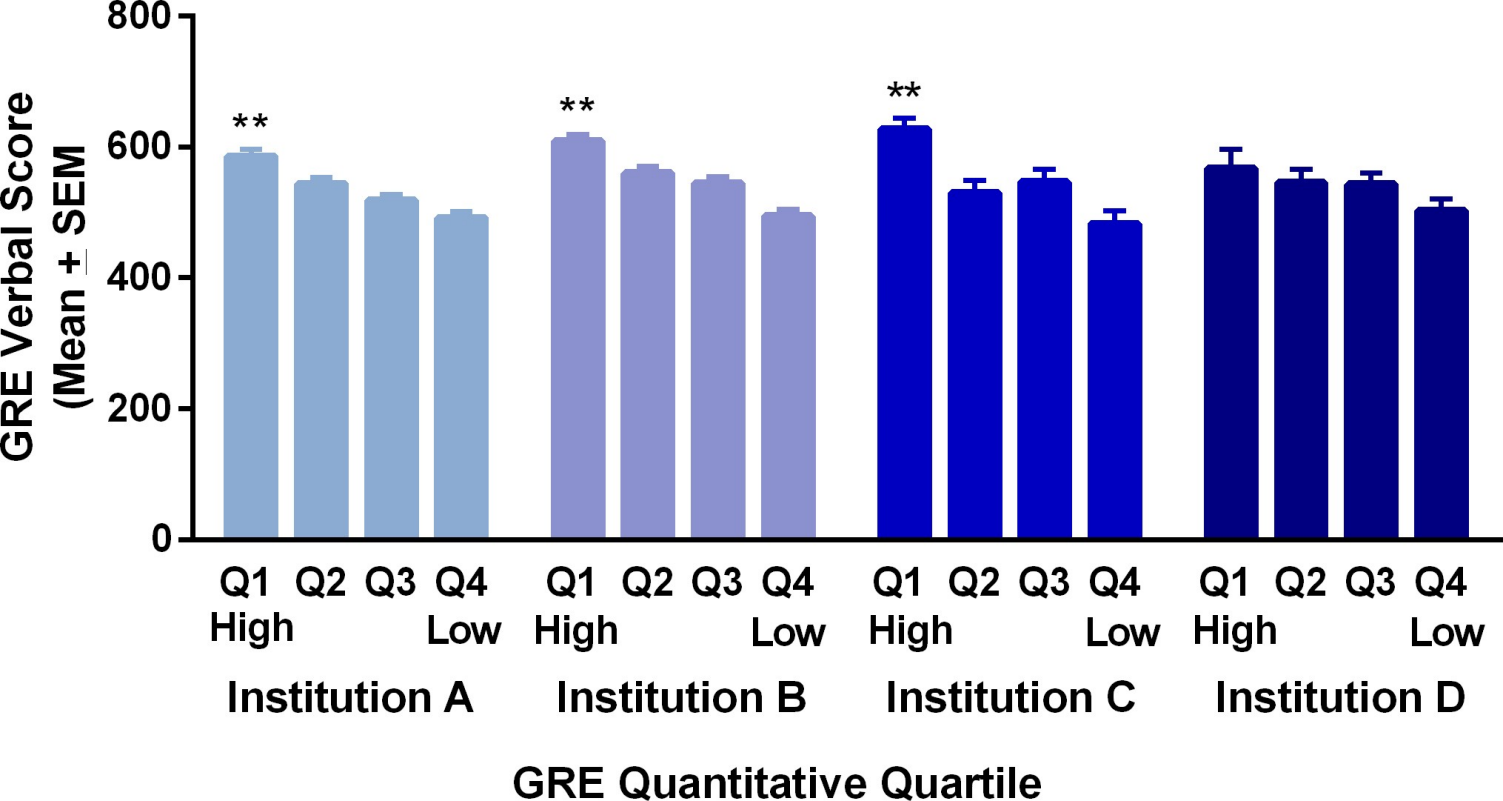
GRE Verbal scores for men with GRE Quantitative scores in different quartiles (Q1-Q4) in four state flagship universities.

We hypothesized that men in the lowest quartile of GRE Q scores might have high GRE V scores that would provide them some advantage in PhD completion, but found that this was not the case. Instead, men with the highest GRE Q quartile scores also had the highest GRE V scores for all institutions (not significantly different in Institution D, the smallest institution) (Fig 3).

We also found that there were no differences in time to degree for men or women based on either GRE V or GRE Q quartile rankings (Table 5).

**Table 5:** Time to degree related to GRE Verbal and Quantitative quartile scores for women and men in a group of 1805 U.S. STEM doctoral students (citizens or permanent residents) from four state flagship institutions.

**Women**				
**GRE Verbal Scores** **Range**	**Quartile 1 (High)** **610–800**	**Quartile 2** **550–600**	**Quartile 3** **470–540**	**Quartile 4 (Low)** **240–460**
**Time to Degree (Years)**				
**Mean**	5.87	5.81	5.74	5.98
**SEM**	0.15	0.13	0.14	0.16
**N**	111	113	111	115
**Men**				
**GRE Verbal Scores** **Range**	**Quartile 1 (High)** **620–800**	**Quartile 2** **550–610**	**Quartile 3** **480–540**	**Quartile 4 (Low)** **250–470**
**Time to Degree (Years)**				
**Mean**	6.19	5.75	5.88	5.88
**SEM**	0.18	0.12	0.15	0.14
**N**	165	164	158	165
**Women**				
**GRE Quantitative Scores** **Range**	**Quartile 1 (High)** **750–800**	**Quartile 2** **680–740**	**Quartile 3** **610–670**	**Quartile 4 (Low)** **290–600**
**Time to Degree (Years)**				
**Mean**	5.83	5.82	5.83	5.94
**SEM**	0.13	0.15	0.16	0.17
**N**	113	112	109	109
**Men**				
**GRE Quantitative Scores** **Range**	**Quartile 1 (High)** **780–800**	**Quartile 2** **730–770**	**Quartile 3** **670–720**	**Quartile 4 (Low)** **200–660**
**Time to Degree (Years)**				
**Mean**	5.86	5.94	5.87	6.02
**SEM**	0.15	0.13	0.14	0.18
**N**	143	184	165	160

Consistent with previous work [17], we found that over 70% of women and 75% of men enrolled in engineering doctoral programs in our sample had GRE Q scores of 700 or above. Table 6 shows that there were no significant gender differences in either GRE V or GRE Q scores for students enrolled in engineering PhD programs, but men in non-engineering STEM fields had significantly higher GRE Q scores than women counterparts. Finally, GRE V scores for men in engineering were significantly lower than for men in non-engineering STEM programs.

**Table 6:** GRE V and Q scores (Mean ± SEM) for women and men in engineering or non-engineering STEM fields.

	GRE V	GRE Q
**Engineering Women (n = 115)**	527.9 ± 9.6	718.7 ± 6.1
**Engineering Men (n = 257)**	516.4 ± 6.2^a^	729.7 ± 3.7
**Non-Engineering STEM Women (n = 652)**	534.4 ± 4.0	662.8 ± 3.4^b^
**Non-Engineering STEM Men (n = 781)**	549.9 ± 3.9	700.6 ± 3.1^c^^,^^d^

^a^Significantly lower than scores of men in non-engineering STEM; p<0.0001.

^b^Significantly lower than women and men in engineering; p<0.0001.

^c^Significantly higher than women in non-engineering STEM fields; p<0.0001.

^d^Significantly lower than men in engineering; p<0.001.

Table 7 shows that for both men and women, GRE V and Q scores were similar for those who completed and those who left engineering PhD programs. GRE Q scores were also similar for women who completed engineering PhD degrees and those who left. In contrast, men who left engineering doctoral programs had significantly **higher** GRE Q scores than those who completed degrees. Completion rates for women did not differ from those for men (60.9% vs. 64.6%; Chi-Squared test).

**Table 7:** GRE Verbal (GRE V), GRE Quantitative (GRE Q) scores (mean ± SEM) for women and men who completed engineering PhD degrees or left without PhD degrees.

		GRE V	GRE Q
**Women Who Completed Engineering PhD Degrees**	**Mean**	528.9	724.4
	**SEM**	12.3	7.4
	**Percentile**	64	70
	**N**	70	70
**Women Who Left without PhD**	**Mean**	526.4	709.8
	**SEM**	15.8	10.4
	**Percentile**	63	67
	**N**	45	45
**Men Who Completed Engineering PhD Degrees**	**Mean**	516.1	723.0
	**SEM**	7.5	4.9
	**Percentile**	61	70
	**N**	168	168
**Men Who Left without PhD**	**Mean**	516.1	741.8*
	**SEM**	9.4	5.0
	**Percentile**	61	74
	**N**	89	89

Percentile rankings of scores were approximated based on data in [23]

*Significantly higher than scores of men who completed degrees; p<0.05

**Fig 4:**
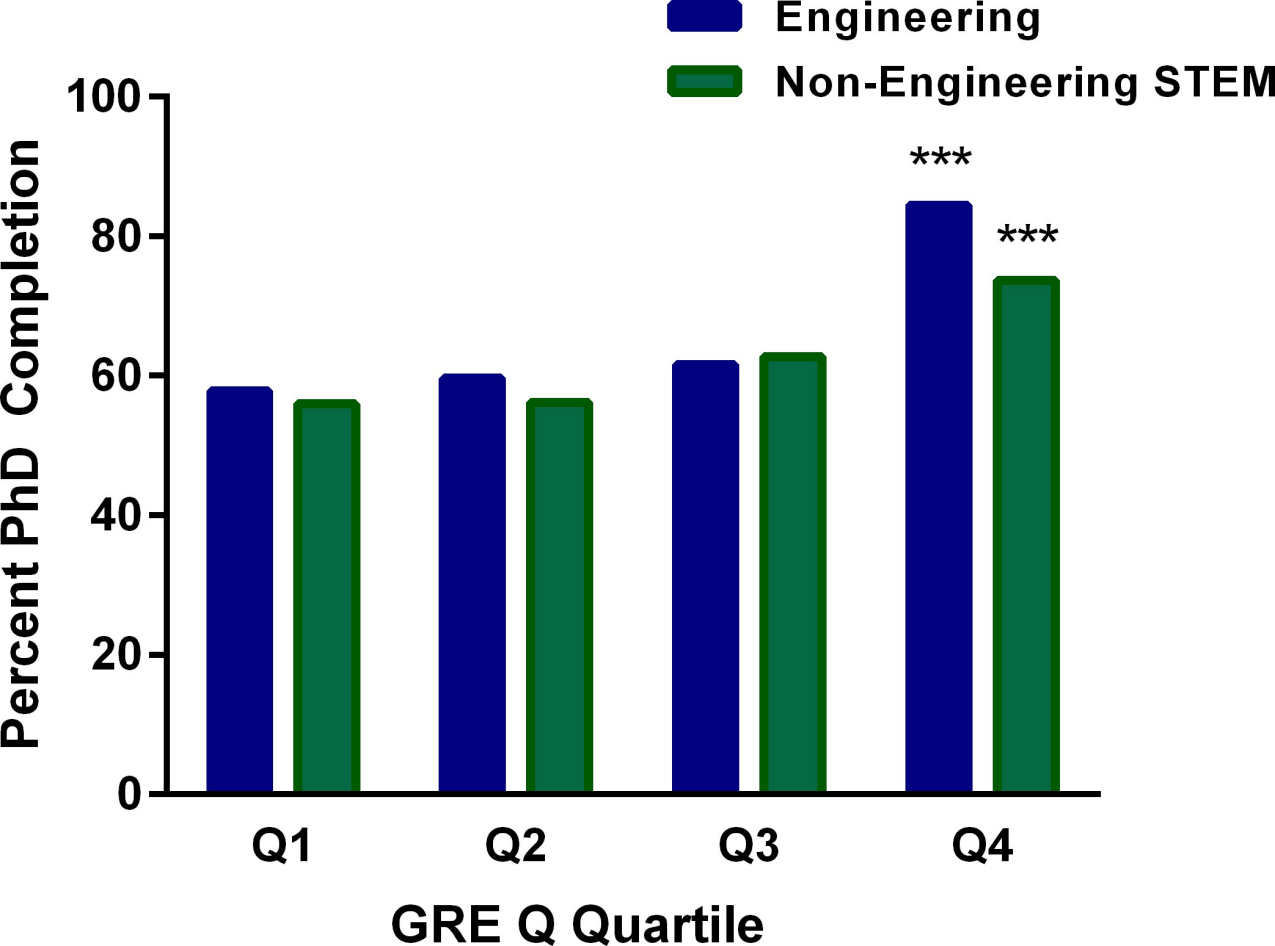
Relationships between rated of engineering and non-engineering STEM PhD completion and GRE Q score quartile (Q1 highest quartile) for men. Ranges and means (±SEM) of GRE Q quartiles for male engineering students were: Q1 = 780–800, mean = 792.8±0.7; Q2 = 750–770, mean = 759.8±0.8; Q3 = 710–740, mean = 725.2±1.0; Q4 = 390–700, mean = 654.4±3.9. Those for male non-engineering students were: Q1 = 780–800, mean = 792.7±0.5; Q2 = 730–770, mean = 750.3±0.9; Q3 = 670–720, mean = 698.0±1.1; Q4 = 200–660, mean = 597.0±3.8. ***Significantly different from completion rates of those in the higher quartile; p<0.0001 (Chi-Squared test).

We divided men into quartiles based on GRE Q scores to further investigate our finding that men who completed PhD degrees in engineering had lower GRE Q scores than men who left their programs. We also compared these men with men in non-engineering STEM programs. As shown in Fig 4, men in the **lowest** quartile of GRE Q scores in both engineering and non-engineering STEM doctoral programs finished at **higher** rates than those in the highest quartile. This is despite the fact that scores of those in the latter group averaged 140 points higher.

## Discussion

Our findings provide strong evidence that GRE scores are not predictive of STEM doctoral degree completion for U.S. and Permanent Resident students. In addition, our data demonstrate the importance of considering women and men separately when studying the relationship between GRE scores and PhD completion. We found that GRE Q scores did not predict PhD completion for women in STEM programs and that, unexpectedly, GRE Q scores were **higher** for men who **left** than for those who completed PhDs. When we examined this finding more closely, we saw that men with GRE scores in the lowest quartile finished at higher rates than any other group, a pattern seen in each of the four institutions. This is particularly surprising because men in the lowest quartile had GRE Q percentile scores averaging approximately 34, and those in the highest quartile had percentile scores averaging 91. It is also notable that GRE scores did not predict time to degree or foretell who would leave during or after the first year. Finally, in engineering, a field in which mean GRE Q scores of admitted students are higher than in other fields [17], men in the lowest quartile for GRE Q scores completed at a rate 25% higher than those in the highest quartile. Overall, our data suggest that if we consider program completion to be the most important index of success, the practice of relying heavily on GRE scores [7, 17] for selecting STEM doctoral students needs to be reexamined.

The Educational Testing Service, the organization that prepares and administers the GRE, advises against having “cut offs” for GRE scores [8], but there is evidence that the practice continues [7]. In our study, we found that in each of the four institutions, women who were enrolled in STEM PhD programs had GRE Q scores that averaged 40 points lower than men (but women completed at rates similar to those of men). These data might be used to support the idea that admissions committees were ignoring GRE Q scores and, therefore, the scores do not represent a source of bias. But, another interpretation is that GRE Q scores may have restricted the number of women admitted because there were fewer women in the pool who had “acceptable” scores as suggested previously [17]. This is especially concerning in fields wherein high GRE Q scores are formally or informally required and women are severely underrepresented [17].

Indeed, our data suggest that GRE Q scores likely had a limiting effect on participation of women in engineering just as they do in physical sciences [17]. GRE Q scores of men and women enrolled in the engineering programs in our sample did not differ significantly, and over 70% of all students enrolled scored at least 700. Data presented by Miller and Stassun [17] suggest that less than 40% of women, but nearly 65% of men who apply to engineering programs score at or above 700. Therefore, the pool of women with scores above 700 was significantly smaller than for men, a factor that may contribute to the finding that women made up less than a third of the engineering doctoral student group in our study. This is of concern because the percentage of U.S. women who earn engineering doctorates has been below 25% over the past 10 years [24]. If a goal of the country is to significantly increase the number of U.S. engineers and to achieve gender parity in the field, it seems reasonable to remove the GRE Q score as an obstacle.

It is particularly troubling that GRE Q scores appear to play such a large role in STEM doctoral admissions decisions because our data show that they do not predict PhD completion for women STEM students and for men they are **negative** predictors. In fact, our current findings suggest that it is not just women who may be excluded, but also talented men who score below 600 on the GRE Q. This group finished at rates far above other groups, suggesting that they have abilities not predicted by GRE scores but key to STEM PhD completion. It was beyond the scope of this project to probe differences that may explain our findings, but we ruled out the possibility that males with low GRE Q scores had high GRE V scores that might be an asset to them. It will now be important to determine what characteristics persuaded admissions committees to accept these men with GRE Q scores in the lowest quartile. We can then study whether these characteristics play a role in STEM PhD degree completion and could be used in admission assessments to identify untapped talent.

Our study is the first to show that GRE Q scores are negative predictors of degree completion for men in STEM, but others have reported similar findings in data not disaggregated by gender. In a large meta-analysis that included 1055 students in life sciences, researchers found a negative correlation between GRE Q scores and degree attainment in that discipline [18]. Others found that GRE Q scores for students who graduated in applied sciences or life sciences were approximately 30 points lower than for those who did not finish [25, 26]. In a study of 340 doctoral students in a group of biology, chemistry and psychology departments, GRE Q scores of students who withdrew were 21 points higher than those who completed [10]. Unfortunately, it was not clear that the difference was statistically significant, and the sample included both men and women. In addition to these studies suggesting that GRE Q scores may be negative predictors of STEM PhD completion, others found that neither GRE Q or V scores of doctoral students differed between those who leave PhD programs and those who progress beyond the third year [19] or who complete programs [20, 21]. It should be noted that one meta-analysis of graduate students not disaggregated by gender, degree type or discipline reported a weak positive correlation between GRE (total) scores and degree completion [27].

The Educational Testing Service publications suggest that GREs are best suited to predict first-year graduate GPAs [10]. This might be relevant to the selection process if GRE scores predict who will fail first-year courses and leave STEM PhD programs during or after the first year. On the contrary, we found that neither GRE V nor GRE Q scores of males or females differed between students who completed PhD degrees and those who left during the same calendar year that they entered. We also found no differences in time to degree based on GRE V or GRE Q quartile scores for either gender, consistent with previous findings of others [21]. Although we did not examine any other indices of success in STEM PhD programs related to GRE scores, Hall *et al*. [20] found that neither GRE V nor GRE Q scores predict the number of first author publications. Moneta-Koehler *et al*. [21] found that GRE V scores were moderate predictors of first semester grades, graduate GPAs and of better subjective faculty evaluations of some aspects of students’ performance. However, these predictions did not translate to differences in time to degree, passing qualifying exams, numbers of conference presentations, or numbers of individual fellowships or grants [21].

The cost of an admission system that is not effective in identifying successful STEM doctoral students goes beyond limiting the number of potential contributors to the innovation economy; it has severe financial consequences to the institutions and the nation. In our cohort of 1805 students, 703 did not complete the doctoral degree and 102 left during the same year they enrolled. Of the 601 students who left after the first year, the average time to leaving was approximately 3 years for both men and women. The annual cost of training students in the four institutions in our study averaged $58,000 per student. Thus, the cost of attrition for those who left during the first year was $5.9 million. For those who stayed for 3 years, the cost approached $105 million. This means that the cost of attrition for the five-year cohort in our study averaged $22.2 million/year. The cost may be significantly higher because those who left after three years likely obtained masters’ degrees. Although this may not be considered a true loss to the U.S. STEM workforce, it may be a revenue loss to institutions that charge tuition and fees to students seeking STEM masters’ degrees, but waive these charges and provide stipends for students seeking PhD degrees. If we apply these calculations to the national cost of attrition for approximately 13,000 students from each entering cohort (assuming a 59% non-completion rate and approximately 18,000 completing STEM PhD degrees [1]), the cost is between $1 billion and $3 billion per cohort. It should be noted that these calculations do not include the potential value of papers, patents and contributions to the teaching mission created by graduate students who did not finish. Also not considered in these calculations are the personal investments of students who do not complete STEM PhD programs and their families, or the time and resources faculty and staff invest in these students.

Over the years and through multiple iterations of the GRE, there have been strong data-based appeals, many from faculty members, to stop using the test in the STEM admissions process [9, 16, 17, 19–21, 28, 29]. In addition, the National Science Foundation no longer requires students to report GRE scores in fellowship applications and the National Institutes of Health does not ask training grant recipients to report GRE scores of their trainees. Still, despite assertions to the contrary, admissions committees continue to rely to a great extent on the GRE [30], particularly on the GRE Q that is arguably the most biased portion of the exam [17]. In addition to erroneously viewing the GRE as predictive of PhD completion, faculty members have numerous, wide-ranging, and largely anecdotal reasons for the strong attachment to the GRE. One of the main problems may be that there are few exemplars of successful students with low scores if most of those chosen have high GRE scores. When a high scorer leaves, faculty accept that “it wasn’t right for him/her”, but if a low scorer leaves, faculty suggest that it was predictable based on GRE scores. In addition, most STEM faculty who are currently in academia necessarily did well on GRE exams or they would likely not have been admitted. Thus, they assume the test was predictive. Finally, a pragmatic reason for relying on GRE scores to identify students for admission is that it speeds up the process, particularly in programs with a large number of applicants.

In summary, this study provides convincing evidence that GRE scores are not predictive of STEM PhD completion for U.S./Permanent Resident students at state flagship research institutions. In addition, relying on the GRE Q is likely to exclude talented students with scores below an arbitrarily defined “acceptable” score, but who have other characteristics that are better predictors of success. Considering the high cost of attrition and its impact on future leadership for the U.S. STEM workforce, it seems prudent to reconsider the role of GRE scores in the STEM PhD selection process. If we can identify the characteristics that motivated admissions committees to overlook GRE scores of men in the lowest quartile, we can study whether these characteristics contributed to the high completion rates of this group of students. In doing so, perhaps we can develop more inclusive and predictive STEM doctoral admissions processes.

## Supporting information

S1 File De-identified data set used for the analyses described herein. (XLSX)
